# Extensive surgical resections for rare pleural neoplasms: a single-center experience with a yolk sac tumor and synovial sarcoma

**DOI:** 10.1186/s12957-024-03367-9

**Published:** 2024-04-16

**Authors:** Tom Vandaele, Jan Van Slambrouck, Patrick Schöffski, Herlinde Dumez, Birgit Weynand, Raf Sciot, Annalisa Barbarossa, An-Lies Provoost, Kristof Van de Voorde, Yves Debaveye, Sofian Bouneb, Philippe Nafteux, Laurens J. Ceulemans

**Affiliations:** 1 Department of Thoracic Surgery, University Hospitals Leuven, Leuven, Belgium; 2 Department of Chronic Diseases and Metabolism, Laboratory of Respiratory Diseases and Thoracic Surgery (BREATHE), KU Leuven, Leuven, Belgium; 3 Department of Oncology, University Hospitals Leuven, Leuven, Belgium; 4 Department of Oncology, Laboratory of experimental Oncology, KU Leuven, Leuven, Belgium; 5 Department of Pathology, University Hospitals Leuven, Leuven, Belgium; 6 Department of Imaging and Pathology, Laboratory of translational cell and tissue research, KU Leuven, Leuven, Belgium; 7 Department of Perfusion, University Hospitals Leuven, Leuven, Belgium; 8 Department of Intensive care, University Hospitals Leuven, Leuven, Belgium; 9 Department of cellular and molecular medicine, Laboratory of Intensive care, KU Leuven, Leuven, Belgium; 10 Department of Anesthesiology, University Hospitals Leuven, Leuven, Belgium; 11 Department of cardiovascular science, Laboratory of anesthesiology and algology, KU Leuven, Leuven, Belgium

**Keywords:** Pleural yolk sac tumor, Pleural synovial sarcoma, Hyperthermic intrathoracic chemotherapy, Pleural tumor

## Abstract

**Background:**

Pleural neoplasms are rare and can be subdivided into pleural metastasis and primary pleural neoplasms. Non-mesothelioma primary pleural neoplasms are a diverse group of extremely rare pathologies.

**Case presentation:**

In this case series, we describe the presentation and management of two rare primary pleural neoplasms. A first case describes a primary pleural yolk sac tumor treated with neoadjuvant chemotherapy, extended pleurectomy decortication, and hyperthermic intrathoracic chemotherapy. In a second case we describe the management of a primary pleural synovial sarcoma by neoadjuvant chemotherapy and extrapleural pneumonectomy. A complete resection was obtained in both cases and the post-operative course was uncomplicated. No signs of tumor recurrence were noted during follow-up in the first patient. In the second patient a local recurrence was diagnosed 6 months after surgery.

**Conclusion:**

Neo-adjuvant chemotherapy followed by extensive thoracic surgery, including hyperthermic intrathoracic chemotherapy, is a feasible treatment strategy for non-mesothelioma primary pleural neoplasms, but careful follow-up is required.

## Background

Malignant pleural neoplasms are rare oncological diseases with the vast majority being pleural metastasis [1]. Among primary pleural tumors, malignant mesothelioma (MM), although rare, is most frequently encountered [1, 2]. Other primary pleural malignancies comprise a rare and diverse group of diseases, e.g. pleural desmoid tumor, malignant solitary fibrous tumor, desmoplastic small round cell tumor, Askin tumor, pleuropulmonary blastoma, or primary pleural lymphoma. These are due to their rarity mainly described in case reports or case series [1–3]. Differential diagnosis of pleural neoplasms is further complicated by a variety of benign pleural neoplasms including solitary fibrous tumors, lipomatous tumors, adenomatoid tumors, calcifying fibrous tumors, simple mesothelial cysts, multicystic mesothelioma, and schwannoma [1]. Because MM is by far the most frequent primary pleural neoplasm representing 90% of all primary pleural tumors, clear guidelines for its treatment are available [2, 4]. However, for non-mesothelioma primary pleural tumors there is a lack of robust evidence to guide therapeutic decision-making.

In this case series, we report the management, surgical procedure, and short-term outcome of two exceptionally rare cases of primary pleural tumors. The first patient suffered from a primary pleural yolk sac tumor which has, to our knowledge, never been described in a living patient. The second patient had a primary pleural synovial sarcoma. Both patients were treated with neo-adjuvant chemotherapy and extensive surgical resection.

## Case presentation

### Primary pleural yolk sac tumor treated with induction chemotherapy, extended pleurectomy decortication, and hyperthermic intrathoracic chemotherapy

**Fig. 1:**
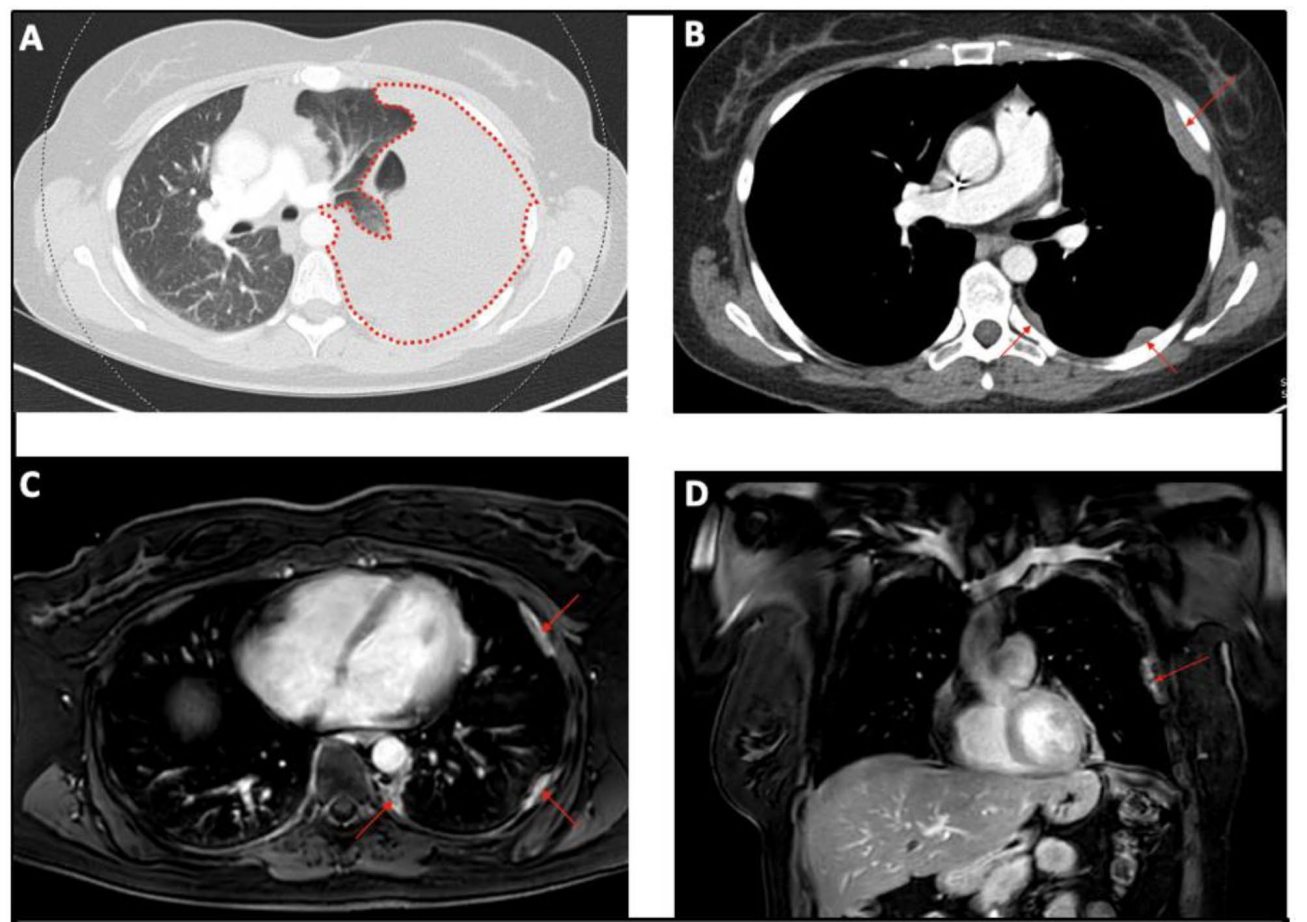
(**A**) Chest CT showing a large left sided pleural effusion; (**B**) Chest CT after thoracocentesis revealing multiple pleural nodules; (**C**) Preoperative chest MRI showing residual pleural disease post-chemotherapy; (**D**) Preoperative chest MRI showing residual pleural disease post chemotherapy

**Fig. 2:**
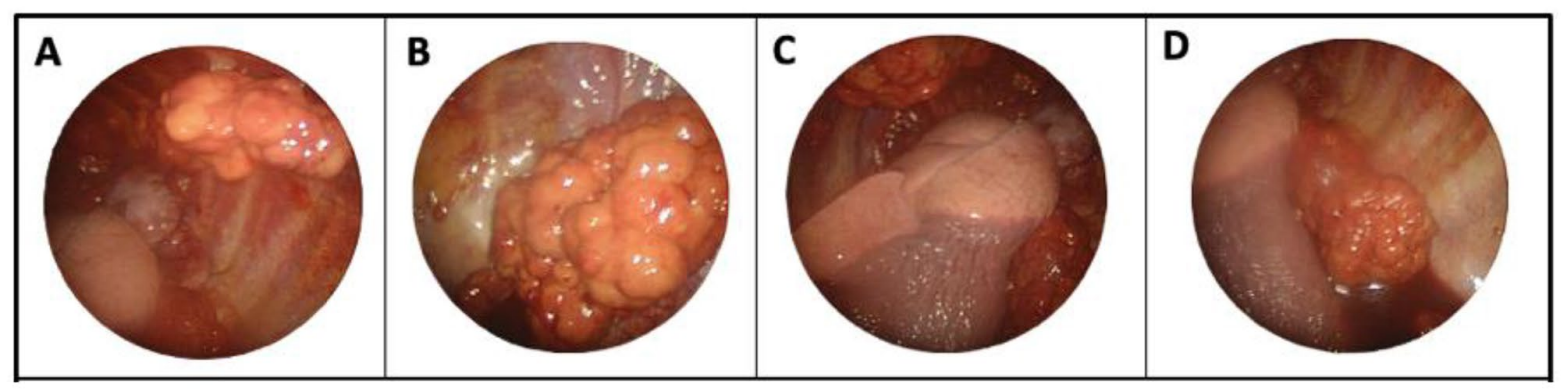
(**A**, **B**, **C**, **D**) Pleural yolk sac tumor on thoracoscopy

**Fig. 3:**
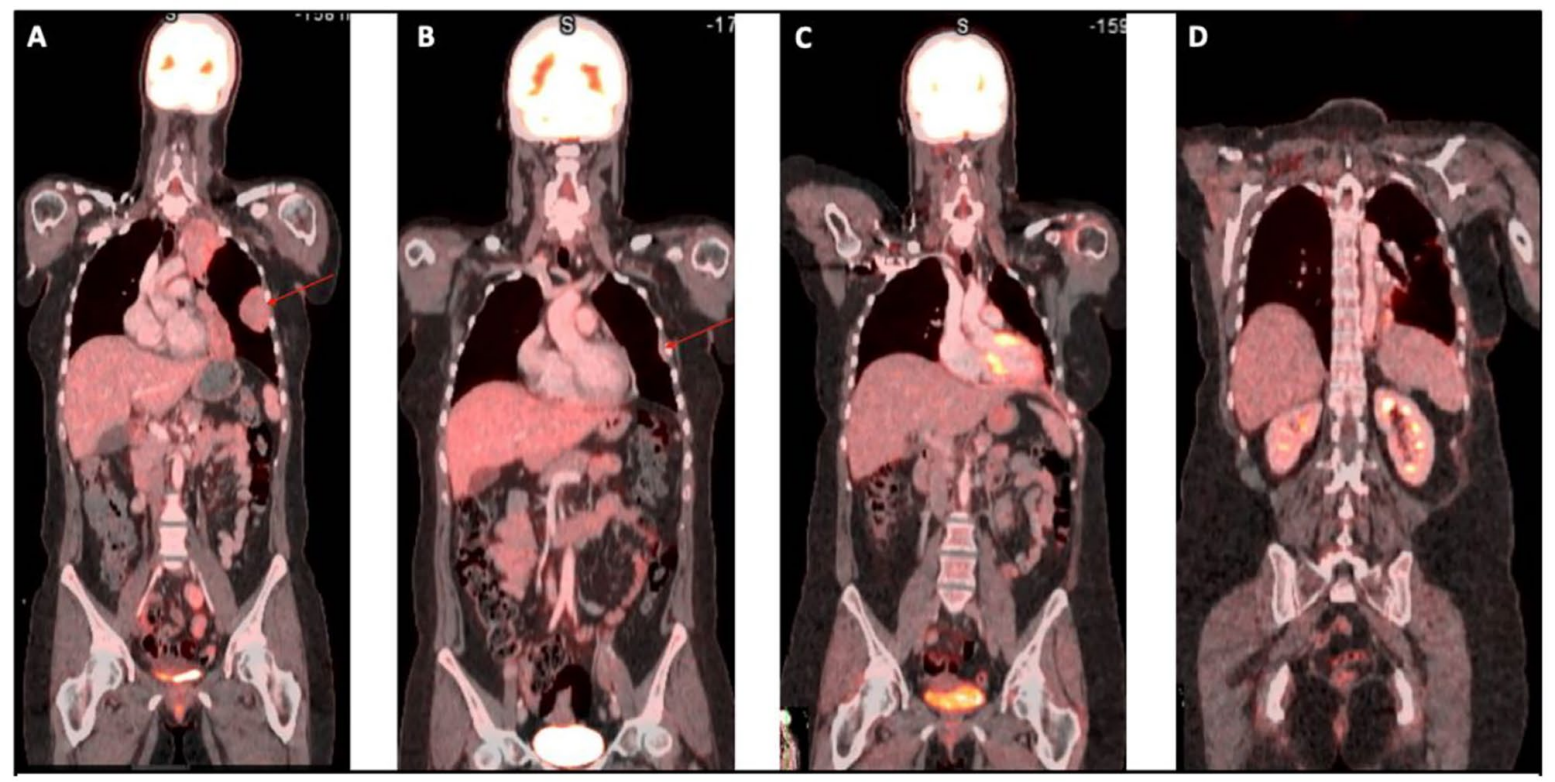
(**A**) PET-CT prior to chemotherapy; (**B**) PET-CT after neoadjuvant chemotherapy; (**C-D**) PET-CT 6 months after surgery

A 37-year-old white female was referred to our center with worsening dyspnea (NYHA II-III), chest pain during breathing, epigastric pain, a weight loss of 5 kg over 6 months, and nocturnal hyperhidrosis. She also had a history of smoking (5 pack years), but no other relevant medical history. An initial work-up for epigastric pain, with abdominal ultrasound and gastroscopy was negative. Because of the worsening dyspnea a chest CT-scan was performed, which revealed a large left-sided pleural effusion and multiple small pleural nodules highly indicative for a malignancy originating from the pleura. (Fig. 1a&b) Initially a thoracocentesis was performed, but histopathological analysis of the pleural fluid was inconclusive. Because of the highly suspicious pleural thickening a uniportal thoracoscopic biopsy was performed to rule out malignancy. Thoracoscopy revealed multiple tumoral nodules on the left parietal and visceral pleura, and left hemidiaphragm. (Fig. 2a-d) Histopathology confirmed the diagnosis of a hepatoid-type pleural yolk sac tumor. Because yolk sac tumors often arise from a gonadal origin, a transvaginal ultrasound was performed to rule out a disseminated ovarian yolk sac tumor [5]. Furthermore, laboratory tests revealed elevated alpha-fetoprotein levels (α-FP) of 68.300 µg/l, further confirming the diagnosis of a yolk sac tumor. To identify any distant metastasis, a PET-CT scan was performed, which only revealed localized hypermetabolic activity in multiple pleural nodules in the left hemithorax. (Fig. 3a)

Etoposide and cisplatin chemotherapy was initiated, resulting in decreased serum alpha-fetoprotein levels (3159 µg/l) and a tumor mass reduction on CT-scan. Due to reduced response after three cycles of etoposide and cisplatin, with a further α-FP decrease to only 2453 µg/l after the last cycle, treatment was switched to paclitaxel, cisplatin, and ifosfamide (TIP-chemotherapy). The TIP-regimen was not tolerated well and associated with hearing loss, bilateral tinnitus, peripheral sensory neuropathy, and eventually neutropenic fever, thrombocytopenia, and anemia (6.8 g/dL). Furthermore, after an initial decrease to 1943 µg/l, α-FP levels started rising again. Therefore, after two TIP cycles, rescue with extensive surgical resection was considered.

A preoperative functional workup was performed, revealing normal pulmonary function tests with a slightly decreased diffusion capacity (FEV1 84% - FVC 89% - TLCO 54%), a normal myocardial perfusion scan and cardiac ultrasound, and a V/Q scan revealing a slight superiority of the right lung (ventilation 60% - perfusion 62%) in comparison to the left lung (ventilation 40% - perfusion 38%). Oncological workup with chest CT and whole-body PET-CT scan showed a reduction of tumoral load and confirmed the absence of extra-thoracic disease (Fig. 3b). Chest MRI visualized the persistence of multiple pleural nodules with involvement of the inferior pulmonary ligament and hemidiaphragm, but without mediastinal or chest wall invasion (Fig. 1c&d).

**Fig. 4:**
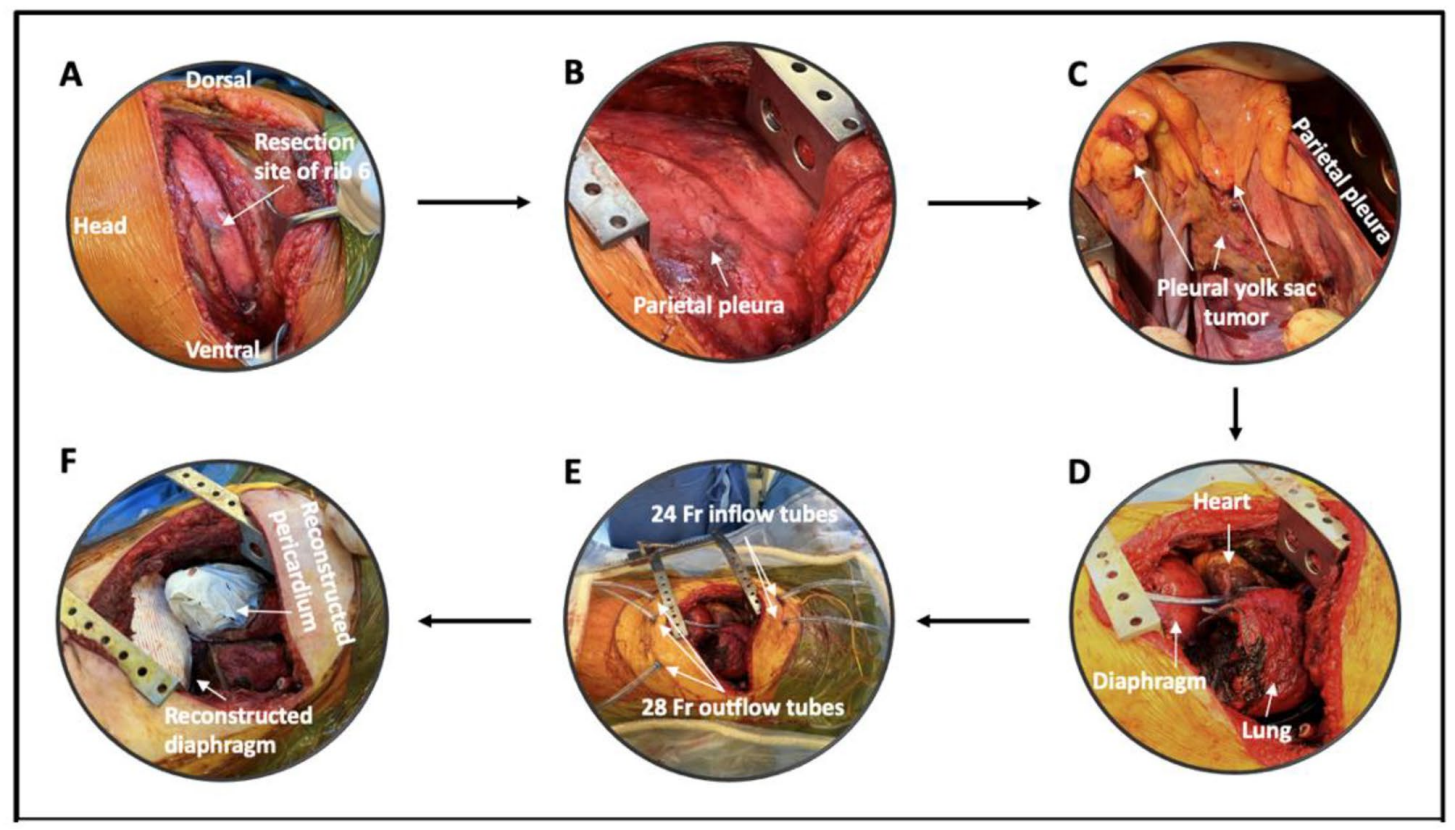
Step by step extended pleurectomy decortication with resection of the left hemi-diaphragm and partial pericardial resection followed by HITHOC and reconstruction. (**A**) Left sided thoracotomy with resection of the 6th rib, (**B**) Extrapleural pleurectomy; (**C**) Visualization of the pleural yolk sac tumor and decortication (**D**) Completion of the extrapleural pneumonectomy with left hemi-diaphragmatic and partial pericardial resection, (**E**) HITHOC setup, (**F**) Final result after diaphragmatic and pericardial reconstruction

To achieve a complete resection, we performed a left-sided extrapleural thoracotomy with 6th rib resection (Fig. 4a&b), followed by an extended pleurectomy decortication (ePD), including the resection of the left hemidiaphragm and a large part of the pericardium. (Fig. 4c&d) Our ePD procedure adheres to a standardized protocol used in all operable mesothelioma cases. This includes a parietal pleurectomy and visceral decortication, followed by meticulous hemostasis and aërostasis, and finally the construction of a neopleura. During the visceral decortication we create a plane between the visceral pleura and the lung parenchyma, then gently peel or strip the visceral pleura outwards while ensuring the integrity of the underlying lung parenchyma. Careful aërostasis is achieved afterwards by suturing major air leaks and constructing a neopleura using an absorbable polyglycolic acid sheet (Neoveil) and polymeric hydrogel sealant (Progel). After complete macroscopic resection, further control of tumor load was pursued with Hyperthermic IntraTHOracic Chemotherapy (HITHOC). The left pleural space was rinsed with 806 mg Oxaliplatin for 45 min. The intrathoracic temperature was kept between 41.6 °C and 42.5 °C. Our HITHOC setup consisted of a RAND Performer HT and a disposable RAND Hang&Go HT basic set containing three 28 Fr outflow drains and two 24 Fr inflow tubes. The total intrathoracic fluid volume was 3500 cc and flow rates of 940 cc/minute were achieved. Eventually the thoracic cavity was rinsed with 3000 cc of Physioneal 40 after 45 min of perfusion. (Fig. 4e) Finally, the diaphragm and pericardium were reconstructed using a Gore-tex patch (Fig. 4f). Postoperative recovery was uneventful, and the patient was discharged from hospital on postoperative day (POD) 26. A brief readmission was needed after presentation with a fever and fatigue caused by a urinary tract infection. Ten months after surgery PET-CT scan showed no signs of recurrence (Fig. 3c&d), and serum α-FP levels decreased to a minimum of 18 µg/l. Currently, the patient is in good physical and mental health, and she has regained weight and functional status.

**Fig. 5:**
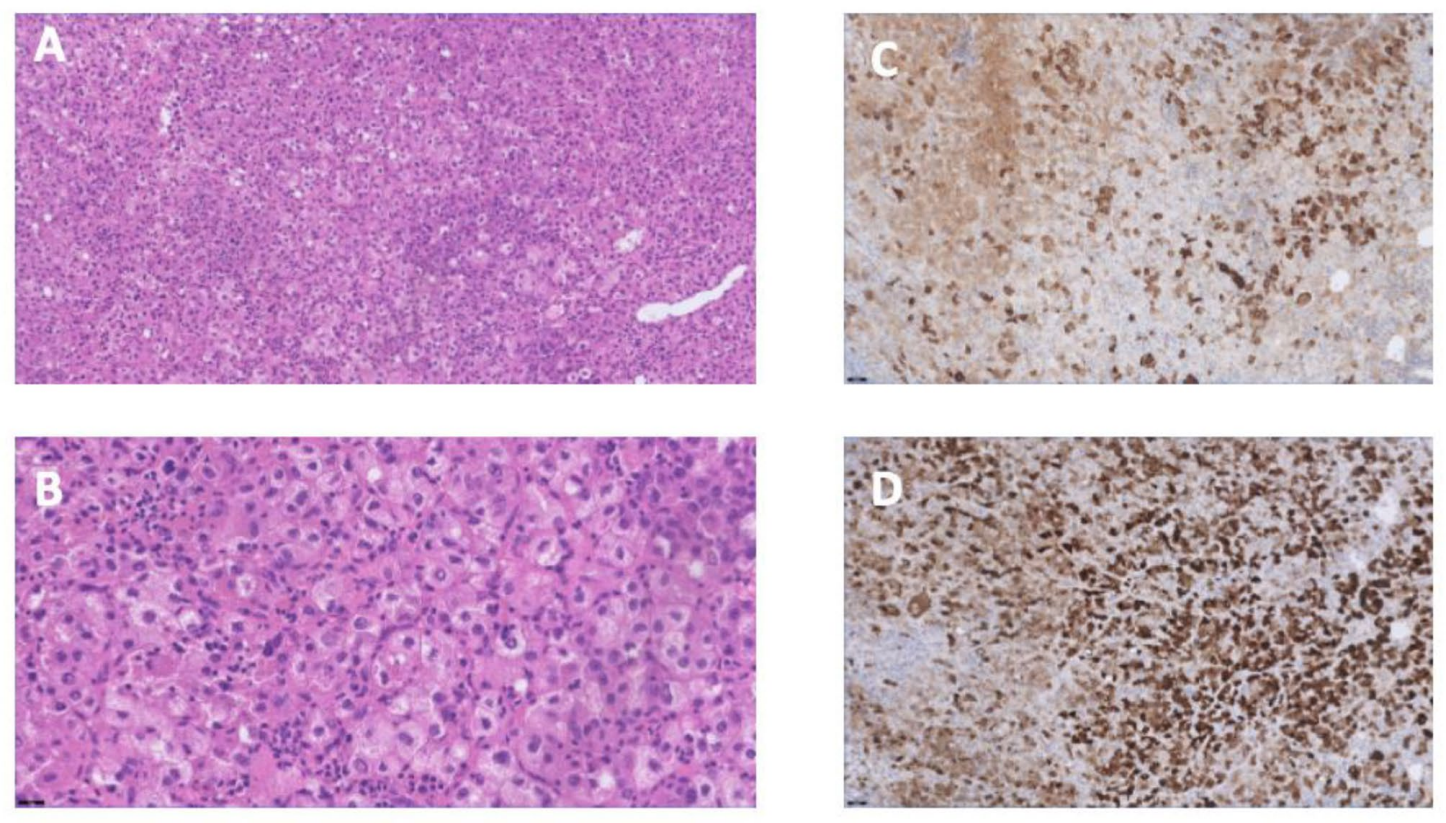
Histopathology of the pleural yolk sac tumor: (**A-B**) hematoxylin and eosin staining of a malignant tumor with a hepatoid aspect. (**C-D**) SALL4 and alfa-foetoprotein staining of the tumor cells proving the germ cell origin. These elements together plead for a hepatoid variant of a yolk sac tumor

The pathology report confirmed a successful and complete (R0) resection of a hepatoid type pleural yolk sac tumor, as evidenced by positive staining for perkeratine, Alpha-Fetoprotein, and Glypican-3. (Fig. 5) However, it is worth noting that SALL4 staining was only weakly positive, and a pure hepatoid growth pattern was observed. Additionally, although a significant decrease in α-FP was noted during neo-adjuvant chemotherapy, the histopathological analysis revealed very little response to the neoadjuvant chemotherapy with extensive residual disease in the resection specimen. The limited response to chemotherapy is rather unusual for a yolk sac tumor. Despite these atypical findings, thorough imaging examinations, and endoscopy with gastric and esophageal biopsies did not reveal any evidence supporting alternative diagnoses such as metastasis from a primary hepatocarcinoma or a hepatoid carcinoma originating from the gastric or esophageal regions. It is possible that different growth patterns, apart from the hepatoid pattern, might have been eliminated by the chemotherapy, challenging final pathological diagnosis.

Due to the rarity, aggressive nature, and ultimately the high risk for recurrence in this young patient, a strict follow-up program is proposed. During the first 2 years post-surgery, a 3 monthly follow-up is scheduled with an alternating control PET-CT and CT scan. After two years, follow-up intervals are extended to 6-months.

### Pleural synovial sarcoma treated with neoadjuvant chemotherapy and extrapleural pneumonectomy

**Fig. 6:**
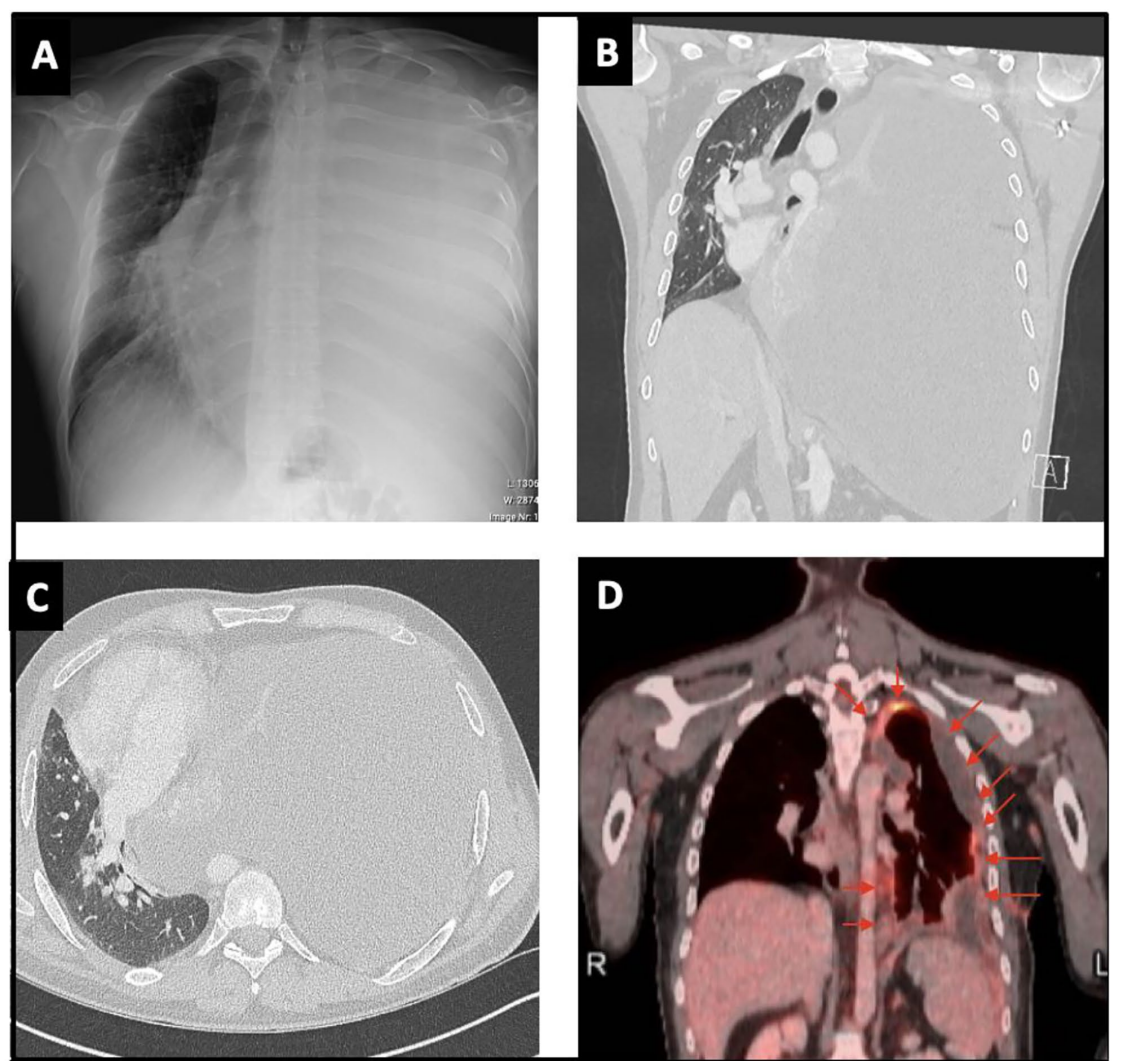
(**A, B, C**) Chest x-ray and CT scan showing a large left-sided pleural effusion with abnormal pleural thickening; (**D**) PET-CT prior to neoadjuvant chemotherapy showing diffuse pleural thickening (partially due to a recent talcage), but without extra-thoracic disease

A 26-year-old male patient was referred to our center with progressive dyspnea (NYHA III), chronic pain in the left hemithorax, inspiratory stridor, and absent breath sounds on the left side. Chest X-ray and CT scan revealed a large left-sided pleural effusion with complete collapse of the left lung and abnormal pleural thickening (Fig. 6a-c). Thoracocentesis drained 2.7 L of pleural fluid in which no cytological evidence for malignancy was found. To exclude tuberculosis, a PCR test and an auramine staining was performed on the pleural fluid but both came back negative. Due to the suspicious nature of the pleural thickening, an exploratory thoracoscopy was performed. Thoracoscopy confirmed diffuse pleural thickening of which multiple biopsies were taken. Microscopic analysis was highly suggestive for a synovial sarcoma. Further histopathological analysis discovered an ss18 rearrangement in 53% of nuclei on FISH analysis, hereby confirming the diagnosis of a pleural synovial sarcoma. Further work-up with whole-body PET-CT scan did not reveal any metastasis. (Fig. 6d)

**Fig. 7:**
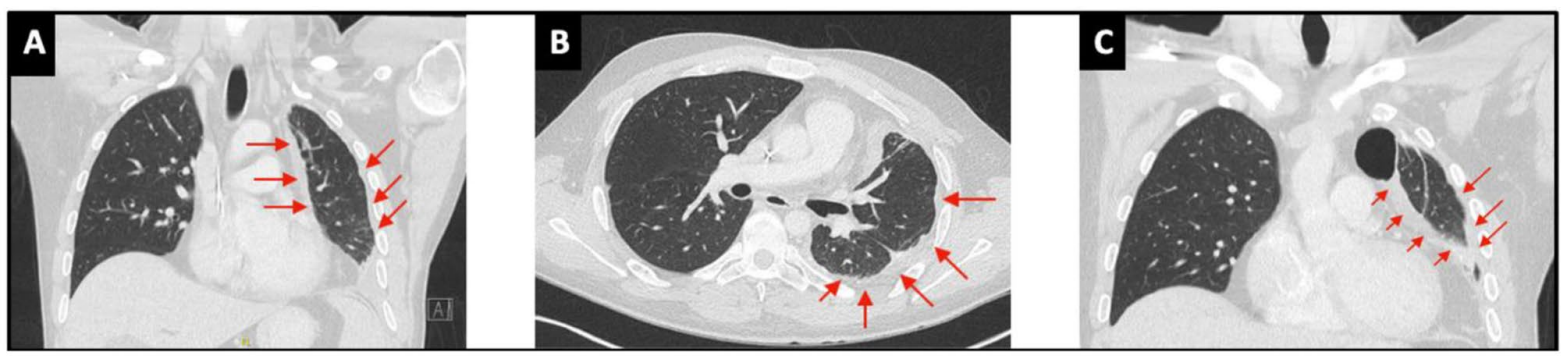
**(A, B, C)** Chest CT-scan after 6 cycles of neoadjuvant chemotherapy showing stable disease

**Fig. 8:**
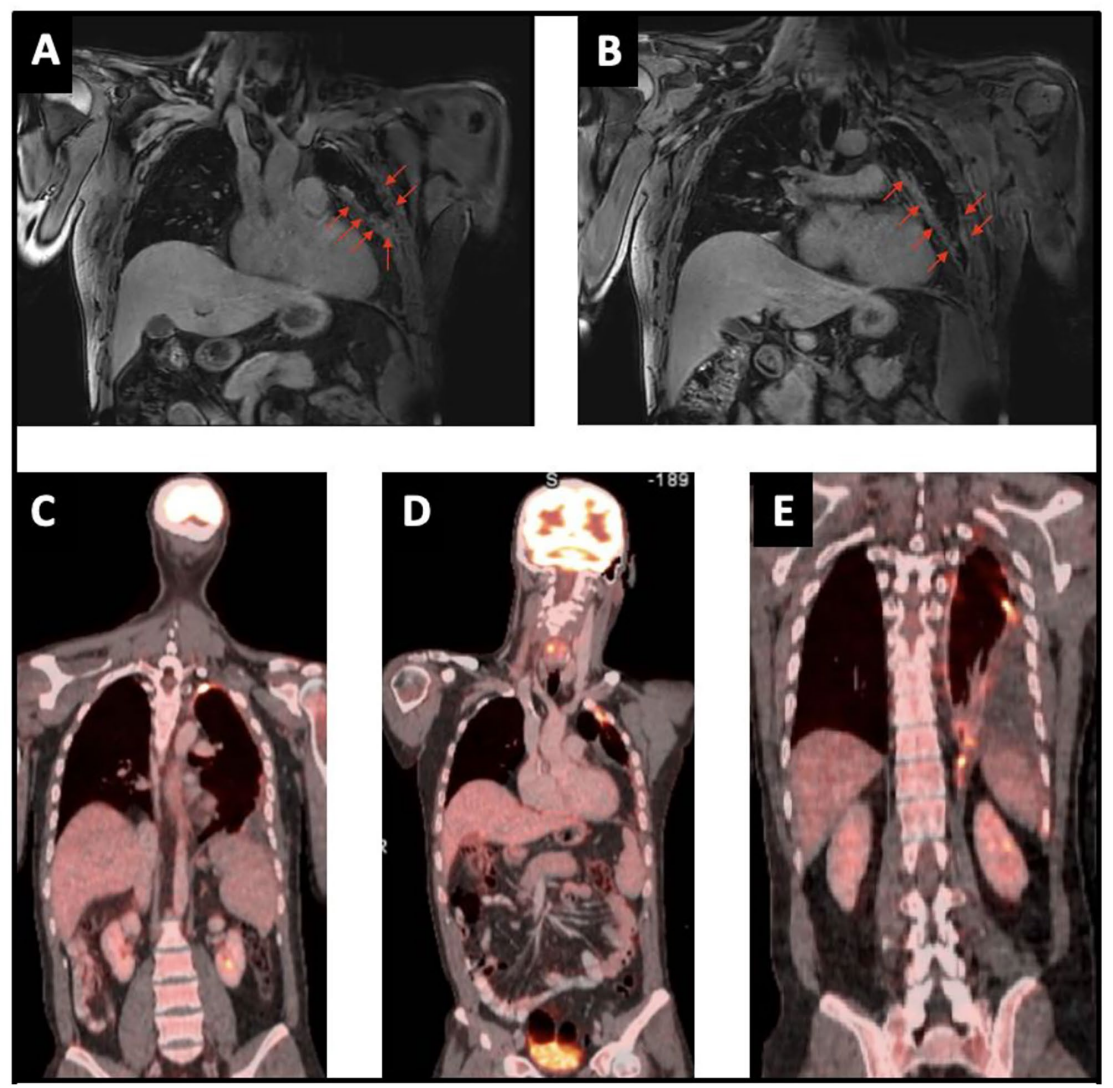
(**A, B**) Preoperative chest MRI showing residual pleural disease after chemotherapy; (**C, D, E**) Preoperative thoracic PET-CT showing residual active pleural disease after chemotherapy

The tumor was initially deemed borderline inoperable, but the recent pleurodesis complicated the evaluation of the extent of pleural involvement on imaging. As such, because synovial sarcomas are generally more chemo sensitive, doxorubicin and ifosfamide chemotherapy was initiated in an attempt to reduce tumoral load, and increase the chance of obtaining a complete resection in the future [6–8]. Chest CT after 6 cycles of chemotherapy showed stable disease but no signs of tumor reduction (Fig. 7a-c). A preoperative functional work-up was performed, myocardial perfusion scintigraphy and cardiac ultrasound were normal. VO_2_ max during cyclo-ergometry was only 43%. Pulmonary function tests revealed a decreased VO_2_ max of 43%, FEV1 of 46%, and FVC of 51%. V/Q scan showed a normal right lung but an important reduction in ventilation (20%) and perfusion (9%) of the left lung. Preoperative oncological work-up with chest CT and MRI showed unchanged tumor dimensions with signs of mediastinal fat involvement but without invasion of the chest wall or mediastinal structures (Fig. 8a&b), PET-CT confirmed residual hypermetabolic pleural disease without arguments for extra-thoracic disease (Fig. 8c-e).

**Fig. 9:**
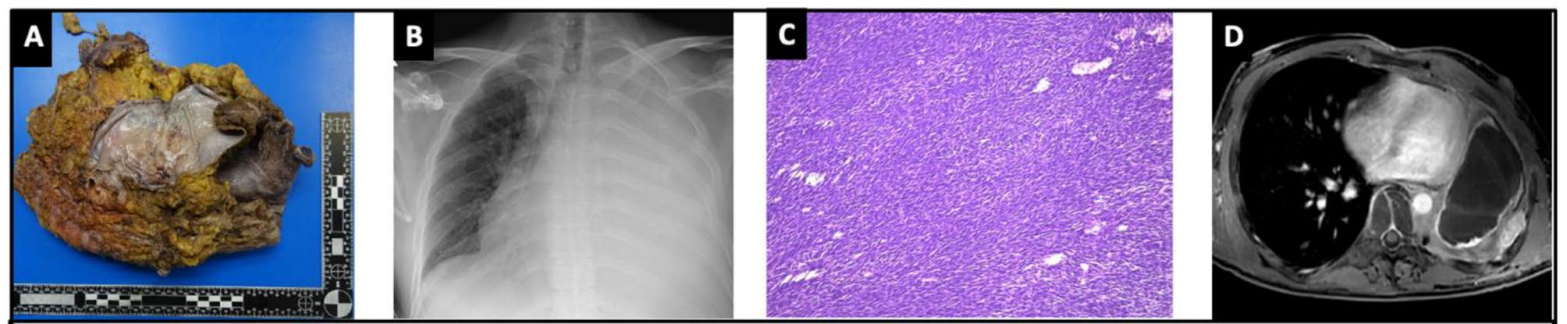
(**A**) Extrapleural pneumonectomy resection specimen; (**B**) Chest x-ray 1 month post extrapleural pneumonectomy; (**C**) Hematoxylin & eosin stain showing the typical fascicular and herringbone growth pattern of a monophasic synovial sarcoma; (**D**) Tumor recurrence on chest MRI after 9 months

After a negative diagnostic laparoscopy to exclude peritoneal involvement and transdiaphragmatic growth, we performed an extrapleural pneumonectomy (EPP) (Fig. 9a&b), with excision of the thoracoscopy ports, a partial pericardial resection, resection of the left hemidiaphragm, and resection of the 6th rib and anterolateral parts of ribs 4 and 5. Our EPP procedure is performed via a standardized protocol, starting with an extrapleural and mediastinal dissection, after which the internal mammary chain and thymus are resected. A minor challenge we encountered during the extrapleural dissection was the presence of multiple adhesions posteriorly where the thoracoscopic biopsies were taken. After this, the diaphragm was resected while leaving the peritoneal lining intact, and a dissection plane around the aorta and esophagus was followed upwards and the pulmonary hilum was transected. After the resection, the thoracic cavity is scrubbed with povidone-iodine to remove any residual malignant cells. Finally, a pericardial and diaphragmatic reconstruction was performed using a Gore-Tex patch. During the several steps of this procedure, multiple frozen sections were analyzed to ensure complete resection.

Histopathological examination confirmed the diagnosis of an R0 resected pleural synovial sarcoma. (Fig. 9d) Surprisingly, in contradiction to what was seen on imaging, the pathological examination revealed a good response to neoadjuvant chemotherapy with only limited viable residual tumor foci present in the resection specimen. The estimated viable tumor load after neo-adjuvant chemotherapy was around 2–3%. The patient experienced an uneventful postoperative recovery and was discharged on POD 8. (Fig. 8c) Due to the young age of the patient, the aggressive nature of the disease, and the high recurrence risk, regular follow-up visits were scheduled with 3 monthly PET-CT scans. During the first follow-up three months after surgery, the PET-CT scan did not show any signs of tumor recurrence. Furthermore, the patient was recovering well both physically and mentally. Unfortunately, PET-CT scan and chest MRI 6 months post-surgery showed a fulminant early tumor recurrence on the chest wall (biopsy proven) for which treatment with trabectidine is ongoning. One year after the operation, the patient is alive.

## Discussion

In this case series we report the management and follow-up of two rare primary pleural neoplasms treated by a multimodal approach consisting of induction chemotherapy and radical surgical resection.

Our first case is a unique report of a primary pleural yolk sac tumor in a previously healthy female. While in literature only one case was published in 2002 with a postmortem diagnosis in a 14-year-old patient, we report, to our knowledge, a first case including diagnosis and treatment of a pleural yolk sac tumor in a living patient [9]. Our approach was based upon the management of other extragonadal yolk sac tumors [10] consisting of neo-adjuvant chemotherapy with bleomycin, etoposide, and cisplatin (BEP-chemotherapy) [10–12]. Bleomycin was omitted in this case due to potential pulmonary toxicity. The response to neo-adjuvant chemotherapy was mainly followed by α-FP levels, as they appear to be a good predictor for the response of yolk sac tumors to chemotherapy, although levels might be influenced by several factors [10]. The initial response to (B)EP-chemotherapy was excellent with a good reduction of α-FP levels from 68.300 µg/l to 3159 µg/l after two cycles. After the third cycle however, a reduced respons was noted with a further reduction to only 2543 µg/l. Because of the reduced tumoral response a switch was made to a TIP-chemotherapy regimen (paclitaxel, cisplatin, and ifosfamide), this choice was based upon its use as a second line chemotherapeutic in testicular germ cell tumors [13, 14] as well as a second line chemotherapeutic in other extra-gonadal germ cell tumors [11, 15]. . Chemotherapy was followed by surgical resection, and HITHOC. In this case we opted for ePD as there were no arguments for lung invasion. Current literature regarding surgical resection of MM also suggests favorable outcomes in selected patients treated with ePD (± HITHOC) compared to EPP [16–18]. 

Concerning HITHOC, there is growing evidence for its use after the resection of pleural neoplasms like MM, disseminated thymoma, and thoracic pseudomyxoma peritonei, and it is generally considered a feasible and safe approach resulting in additive local tumor control [17, 19–23]. Furthermore, hyperthermic intraperitoneal chemotherapy (HIPEC) has previously been described in the treatment of ovarian yolk sac tumors [24, 25]. In our HITHOC protocol we opted for oxaliplatin, based on our previous experience with HITHOC for disseminated thymoma [19, 20], the immediate cytotoxic effect of Oxaliplatin, and it’s favorable side effect profile [19]. In our recent cases of disseminated thymoma and thoracic pseudomyxoma peritonei treated with HITHOC, no relevant HITHOC-related morbidity or mortality was observed and both patients remained free of disease during oncological follow-up [19, 20]. Currently, we cannot compare our results to other cases because this is the very first case of a pleural yolk sac tumor published in literature. However, other cases on pulmonary and mediastinal yolk sac tumors have been published. Generally considered as rare and aggressive, thoracic yolk sac tumors typically exhibit unfavorable outcomes, and treatment usually consists of a combination between neo-adjuvant chemotherapy and/or surgery and/or adjuvant chemotherapy [11]. Notably, in many cases, authors choose a similar approach as in our case, with neo-adjuvant chemotherapy (most often BEP-regimen), followed by complete surgical resection [11, 26–29]. More recently a meta-analysis was published on mediastinal yolk sac tumors revealing a poor prognosis with a median time to survival of 23 months for adult patients, 3-year overall survival of 24.4%, and 5-year overall survival of 23% [30]. On multivariate analysis the main predictors for favorable outcome were: undergoing surgery, receiving chemotherapy, and a more anterior localization of the tumor [30]. Furthermore, the authors claim that the most important cornerstones of the successful treatment of such malignancies are (1) early diagnosis (2) neoadjuvant chemotherapy and (3) surgical resection [30]. 

The second case describes the treatment of a pleural synovial sarcoma in a young adult male. Pleural sarcomas are rare and aggressive tumors, accounting for less than 1% of all primary pulmonary malignancies [31]. The first case of a pleural synovial sarcoma was published in 1996 by Gaertner et al. [32] Since then, few cases have been reported by others [31, 33–43]. A range of treatments have been suggested for pleural synovial sarcoma including extensive surgical resection, (neo)adjuvant chemotherapy (with ifosfamide and doxorubicin), and radiotherapy [33, 38–40, 42–45]. In our case, the patient was initially deemed borderline inoperable due to the diffuse intrathoracic involvement. Although controversial and with limited evidence, an attempt was made at downstaging the tumor with ifosfamide and doxorubicin to facilitate a potential R0 resection in the future [4, 46–48]. While current ESMO guidelines state that the role of neo-adjuvant chemotherapy in the treatment of synovial sarcoma’s remains uncertain, other authors agree that neoadjuvant chemotherapy can offer a benefit in selected cases who were initially not/borderline eligible for resection [7, 47–49]. The choice of chemotherapeutic agents was based upon (neo-)adjuvant and palliative chemotherapy regimens used in other synovial sarcomas [7, 8, 46, 47]. Achieving a complete surgical resection is crucial when treating sarcoma’s because it is directly associated with improved survival outcomes [45]. In our case we opted for a radical resection with an EPP because of the young age of the patient, the favorable V/Q scan, and the limited response to chemotherapy. Adjuvant radiotherapy is most often used when complete resection is not feasible or where residual disease is anticipated after surgery [33, 50]. 

As shown in previous cases of pleural synovial sarcoma’s, the mainstay treatment is complete surgical resection when feasible, often combined with other (neo)-adjuvant treatment modalities. Nonetheless the prognosis of pleural synovial sarcoma’s remains poor, with a median time to recurrence or metastasis of 8.5 months according to a 26-patient case series of pleuropulmonary synovial sarcoma’s [45]. A more recent study with 20 cases reports a 2-year overall survival of 51% and a 5-year overall survival of 22%, a median overall survival of 25 months, and a median disease-free interval of 8.5 months [48]. Notably, as seen in our case, other authors also report a good response of pleural synovial sarcoma’s to neo-adjuvant chemotherapy [51, 52]. 

A variety of options are available when a recurrence of a pleural sarcoma occurs. When feasible, recurrences can be treated with radiotherapy [53] or secondary resections [37, 39, 40, 54]. Adjuvant or salvage chemotherapy can be considered for metastatic disease, local recurrences, or locally inoperable cases [39]. Nonetheless, the exact choice of chemotherapeutic drugs remains a subject of debate [40, 45, 53]. Alternative adjuvant therapies, such as rh-endostatin combined with ifosfamide or pemetrexed, as well as the use of sunitinib as maintenance therapy, have been suggested in the treatment of recurrent disease [36]. In our case an early fulminant local recurrence occurred. Because of the fulminant nature of the recurrence, re-resection or radiotherapy were not considered reasonable options. Therefore we opted for trabecitidine as a second line agent after treatment with Ifosfamide and Doxorubicin [55]. Trabecitidine has shown acceptable results in a retrospective multicenter trial looking at metastatic synovial sarcoma’s, with an overall response rate of 15%, a tumor control rate of 50%, and a median progression free survival of 7 months in responders [55]. Further options in case of recurrent/non-resectable/metastatic disease are dacarbazine and pazopanib [55]. 

HITHOC was not considered in the sarcoma case because of the limited response to systemic chemotherapy. Currently, there are no reports on the use of HITHOC for pleural synovial sarcoma. The first choice of chemotherapeutic drug would be ifosfamide or doxorubicin. However, ifosfamide is not suited due its need for metabolic activation [56]. Doxorubicin on the other hand can be a viable option for HITHOC and has already been successfully administered in HIPEC cases, and in HITHOC for disseminated thymoma and MM [19, 57–60]. 

Repeated cycles of chemotherapy and extensive thoracic surgery affected the physical condition of both patients, requiring intense physical rehabilitation with extended recovery times.

During the follow-up of our first case, no signs of tumor recurrence have been found up to 6 months after surgery, and the patient remained in good general health during follow-up. Our second patient, although asymptomatic and in good general health, experienced an early limited local tumor recurrence 6 months after surgery for which further treatment with trabecitidine is ongoing. Due to the high risk for recurrence in these cases, a strict and multidisciplinary follow-up is essential, with routine PET-CT scans and additional MRI imaging and CT guided biopsies in case of doubt.

Because both cases are rare and at least some parts of the chosen treatment protocols are novel, with limited evidence on the efficacy of certain treatment choices, both patients were extensively informed about the unique therapeutic approach. The potential risks, benefits, and expected outcomes were carefully explained to the patient and informed consent was achieved in both cases. Both cases were also followed by a multidisciplinary oncological advisory board which supported the clinical decision.

## Conclusion

In this case series we describe the treatment of two rare pleural neoplasms, one involving a pleural yolk sac tumor treated with extended pleurectomy decortication and hyperthermic intrathoracic chemotherapy, the other involving a pleural synovial sarcoma treated with an extrapleural pneumonectomy. Our findings confirm that neo-adjuvant chemotherapy (+/- HITHOC) followed by extensive surgical resection is a feasible strategy for non-mesothelioma primary pleural neoplasms, with the potential of favorable short-term oncological outcomes in selected cases. Nonetheless, strict follow-up is crucial due to the high risk for recurrence and the need for timely reintervention.

## Data Availability

No datasets were generated or analysed during the current study.

## Abbreviations

MM: Malignant Mesothelioma
NYHA: New York Heart Association
α-FP: Alpha-fetoprotein
ePD: Extended Pleurectomy Decortication
POD: Postoperative day
EPP: Extrapleural Pneumonectomy
HITHOC: Hyperthermic Intrathoracic Chemotherapy
